# Incidence, etiopathogenesis and pathological aspects of genitourinary tuberculosis in India: A journey revisited

**DOI:** 10.4103/0970-1591.42618

**Published:** 2008

**Authors:** Prasenjit Das, Arvind Ahuja, Siddhartha Datta Gupta

**Affiliations:** Department of Pathology, All India Institute of Medical Sciences, New Delhi, India

**Keywords:** Cervix, endometrium, etiopathogenesis, genitourinary tract, India, kidney, sequel, tuberculosis

## Abstract

**Background:**

Tuberculosis is one of the major health problems in India. Genitourinary tuberculosis comprises 20% of all extrapulmonary tuberculosis, and is the most common extrapulmonary system to be affected by this disease. The recent surge in the incidence of HIV-infected patients in India has further ignited the fury. Though the members of the Mycobacterium species are well identified, the incidence could not be controlled due to its complex etiopathogenesis and genetic background.

**Pathological Spectrum:**

The spectrum of pathological changes of genitourinary tuberculosis is wide, which varies from normal morphology to markedly scarred kidney, bladder, and epididymis with autocystectomy. A thorough knowledge is required to prevent the end-stage complications. The sequel can be detrimental for the patient's physical, behavioral, psychological, and financial health.

**Diagnostic Dilemmas:**

Though culture and polymerase chain reaction are available for the detection of tuberculosis, the sensitivity and specificity varies widely and one should be aware.

**Conclusions:**

A thorough knowledge of epidemiology, immunopathogenesis, spectrum of the disease and the possible sequels, will help better and effective management of the disease.

## INTRODUCTION

The term ‘genitourinary tuberculosis’ was introduced by Wildbolz in 1937, and since then, renal and epididymal tuberculosis were considered together as the local manifestation of the same blood-borne infection.[1] Genitourinary tuberculosis (GUTB) is still a major health problem in many developing countries including India and had been declared by World Health Organization (WHO) as ‘public health emergency’ in 1993.[2,3] In India the estimate of TB is 168/100,000 population/year (WHO 2005 estimates) with an annual incidence of 2.2 million/year (worldwide six million new cases) and an annual death rate of 29/100,000 population/year.[2,3] In comparison to only pulmonary TB, which comprises around 68.4%, the incidence of combined pulmonary-extrapulmonary cases and extrapulmonary TB alone comprise 12% and 20-25% of the total disease burden respectively.[1] Amongst extrapulmonary TB, GUTB accounts for 4% of the load.[3] In comparison to the patient's complaints, the sequel of genitourinary TB is volcanic and requires proper understanding.

## INCIDENCE

### Genital tract tuberculosis

The most common form of extrapulmonary TB is genitourinary disease, accounting for 27% (range, 14 to 41%) worldwide. In India the incidence of genital tuberculosis is nearly about 18%.[4]

#### Female genital tract tuberculosis:

It is estimated that 1% of infertile women, aged between 20-40 years in United States and 18% in India suffer from genital TB.[2] In females the genital organs commonly affected are as follows: fallopian tube (95-100%), endometrium (50-60%), ovaries (20-30%), cervix (5-15%), myometrium (2.5%) and vulva/vagina (1%).[5]

#### Male genital tuberculosis:

Male genital TB is predominantly associated with tuberculosis of the kidney and prostate, seminal vesicle, epididymis, testes as well as scrotum may occasionally be affected.[6]

### Urinary tract tuberculosis

#### In general population:

In India, the incidence of urinary tract TB comprises 4% of the disease burden.[7] In a study by Venkata *et al.*, 69.4% of urinary tract TB was association with dismorphic kidney disease, with an age of occurrence between 25-77 years and a male to female ratio of 33: 3.[8]

#### Tuberculosis in HIV-infected patients:

Currently, amongst the new TB cases detected in India, 5.2% are diagnosed to have HIV (15-49 years) and in an average, 10% of all cases of TB worldwide are HIV-related (1999 data).[9,10]

#### Tuberculosis in post-transplant patients:

The prevalence of post-transplant TB varies from 1% in Germany to 9.5-14.7% in India, with 5-50 times cumulative risk of infection than in the general population.[11]

#### Tuberculosis in children with nephrotic syndrome:

The conventional diagnostic tests, are often unhelpful in these children, and need high index of suspicion, as in a study by Gulati *et al.*, 9.3%, amongst a total of 300 children with nephrotic syndrome had renal tuberculosis.[12]

## ETIOPATHOGENESIS

Tuberculosis is a chronic infection, caused by different species of *Mycobacterium tuberculosis* complex, such as *M. tuberculosis, M. canettii, M. africanum, M. bovis, M. microti, M. pinnipedii, and M. caprae*. Commonly, *Mycobacterium tuberculosis, bovis,* and *africanum* are infectious. While *M. tuberculosis* is the major cause of TB in humans, *M. africanum* sometimes causes pulmonary TB in humans in Africa. Illness occurs either from direct bacterial invasion to any organ in the body or by abnormal immune reactions secondary to mycobacterial products. Tubercle bacilli can remain dormant in tissues and persist for many years.[13] Along with the type of mycobacterial species, duration of exposure, size and infectivity of the strain are also responsible for the difference in infectivity.[1]

### Tuberculosis in vitamin D deficiency

There is sizeable evidence that a fall in serum 25-OH-vitamin D3 level compromises cell-mediated immune defenses, leading to the activation of latent tuberculosis. In a study on Gujaratis in West London, 10-fold increased risk of developing active tuberculosis was described in vitamin D deficiency.[4]

### Sources of genitourinary tuberculosis

At the time of primary TB, the disseminated microorganisms through the blood stream to different organ systems remain dormant in latent foci. In 5-15% of infected patients, these dormant foci break down (liquefaction necrosis and cavitation) causing dispersion of tubercle bacilli.[14] This secondary disease, or reactivation TB, occurs as a consequence of a decreased cellular immunity.

Genitourinary TB is usually caused by reactivation of these dormant organisms, usually within the first two years following the primary infection by *M. tuberculosis* (90-95%) and very rarely (5-10%) by *M. bovis*, where the source of infection is the gastrointestinal tract.[13]

#### TB of female genital tract:

The bacilli reach the genital tract by three principal routes. The hematogenous route (90%), descending direct spread or by lymphatic spread. Primary infection of genitalia rarely may occur from direct inoculation during sexual intercourse with patients with genitourinary tuberculosis.[5] Trans-serosal exudation may give rise to pelvic inflammatory disease and subsequently in extensive pelvic diseases.[14] Very rarely sexual transport has been reported, as 3.9% men with GUTB harbor bacilli in semen.[1]

#### TB of male genital tract:

In men, the sites most commonly involved are epididymis, followed by the prostate. Testicular involvement is less common and usually is the result of direct extension from the epididymis. Tubercular prostatitis usually results from antegrade infection within the urinary tract. Many theories have been postulated to define the precise route of infection to the epididymis. These include - i) Infected urine theory ii) spread via lymphatic system and iii) metastatic spread through the blood stream. Female to male transmission (venereal transmission of TB) is very rare. Testicular involvement is usually as a result of local invasion from the epididymis, retrograde seeding from the epididymis and rarely by hematogenous spread. Involvement of scrotal wall suggests local extratesticular extension of disease process. Male genital tuberculosis usually is associated with renal TB in 60 to 65% cases or with pulmonary TB in around 34% cases.[15]

### TB of urinary tract

In the kidney, hematogenous spread primarily involves the renal cortex and remains dormant. Abnormal host defense mechanism leads to reactivation of these foci with enlargement. Later, the abscess may rupture into the proximal tubule and loop of Henle with eventual development of enlarging, caseating granulomas with papillary necrosis.[7] Spread to the renal pelvis produces pyonephrosis-like lesion, also known as a “cement” or “putty” kidney, which frequently spreads down to the ureters, bladder, or urethra, resulting into ureteric strictures and segmental dilation and obstruction.[7] Tuberculosis of the ureter usually starts in the ureterovesical junction.[1]

### Immune response in tuberculosis

Though *M. tuberculosis* stimulates both the humoral and cellular immune systems, the antibodies are not protective. Activation of cellular immunity blocks and the extent of disease within four to six weeks of initial infection and elicits typical granulomas (also called tubercles), where macrophages are transformed to giant epithelioid cells. “Excessive delayed-type hypersensitivity” with cytolytic T-cell activity, leads to the degeneration of the center of the lesion. In general, CD4^+^ cells (Helper T cells) form large aggregations dominating the granulomas while the CD8^+^ cells (Cytotoxic T cells) are sparse and distributed more toward the periphery of the lesion (immunosurveillance function).[16]

### Role of cytokines in tuberculosis infection

In a study based on a mice model, due to the effect of pro-inflammatory cytokines such as IL-1α′ and TNF-α′ in early infection, there was mild inflammation with low and stable concentrations of PGE_2_, which contributed to an efficient iNOS expression permitting temporal control of the infection.[17]

## PATHOLOGY OF GENITOURINARY TUBERCULOSIS

Amongst all GUTB, kidney alone and kidney with urinary bladder or ureter is affected in more than 70% cases.[7]

### Gross pathology

#### Renal TB:

Kidneys may be involved in two ways, either in the form of miliary TB-multiple cortical white nodules of around 1 mm due to hematogenous spread of bacilli, or cavitary renal TB (localized ascending infection) and predominant medullary lesions.[7,16] The cortical granulomas may remain dormant, asymptomatic, and stable for as long as 10 to 15 years.[16] When they coalesce, cavities are formed, which communicate with the pelvicalyceal system via erosion (moth-eaten appearance on ultrasonography), may rupture or cause part of the papillae to become necrotic, which eventually sloughs out. The end result is a destroyed, defunct calcified kidney (autonephrectomy).[16] At this stage, multiple surface scars are noted on the kidney along with dilated and deformed renal excretory system, filled with caseous necrotic material (pyonephrosis). Later on the only remains may be necrotic material surrounded by fibrous tissue, commonly called ‘cement’ or ‘putty’ or ‘chalk’ kidney.[7]

Diffuse spotty calcification, large calcified abscess or medium-sized calcification causing deformity of calyxes may be noted in collecting system, in 24% of cases. Characteristic calcifications in a lobar distribution are often seen in end-stage TB.[18] The calcified matrix may harbor tubercle bacilli, and if detected radiologically requires surgical excision.[1] Sometimes both kidneys may be slightly enlarged due to amyloidosis or diffuse proliferative glomerulonephritis secondary to TB.[16]

On the other hand, in tubercular interstitial nephritis, the kidney is generally of normal size and shows smooth contour. Even urine culture is sterile. This entity can only be diagnosed by demonstrating the granulomatous involvement in renal interstitium.

#### Ureteral TB:

Ureteral dilatation and a ragged irregular appearance of the urothelium are the first signs of ureteral TB (“beaded” or “corkscrew” ureter). There may be obstruction at the ureterovesical junction or associated tuberculous cystitis and ureteritis.[16] Ureteral shortening and fibrous contraction may give rise to a ‘golf hole’ orifice in the bladder.

#### Urinary bladder TB:

Urinary bladder TB may be induced either by local instillation of BCG, which causes a self-limiting, low-grade, superficial cystitis. Bladder TB affects the mucosa near the ureteral orifice to start with. In advanced infection, the bladder becomes small, irregular, contracted and calcified and eventually may lead to nonfunctional urinary bladder (autocystectomy).[1,19] Rarely fistulas may develop.

### Female genital tuberculosis

#### Fallopian tube:

In early phases, tube diameter is normal and changes are noted mainly in advanced disease, in the form of nodular transformation, mimicking salpingitis isthmica nodosa. Adhesion may occur between ovaries and other pelvic organs with loss of fimbrial structures. Patent ostia along with grossly diseased fallopian tube are often an indicator of tubercular salpingitis.[14]

#### Endometrium:

Diagnosis is often missed in biopsies, as the involvement can be focal. In widespread endometrial TB ulceration, caseous necrosis and hemorrhage can be seen.[14]

#### Ovary:

Affected in 10% cases. Adhesion with the fimbria or formation of unilateral or bilateral adnexal mass can be seen. Gross caseous necrosis in ovaries is uncommon.[5]

#### Cervicitis:

Grossly, the cervix can be normal, ulcerated or may present with a mass mimicking malignancy.[14]

#### External genitalia:

Rarely can involve the vulva in the form of non-healing ulcers.

### Male genital tuberculosis

#### Tubercular epididymitis:

The globus minor is affected alone in around 40% cases, owing to greater blood supply. Bilateral involvement can be noted in 34% cases.[1] Grossly, the vas or epididymis may be beaded. Rarely, discharging sinuses may develop.[19,20]

#### Prostate, testes, penis, urethra:

Prostate may enlarge and show signs of inflammation. Gross caseous necrosis is often identifiable. Testicular TB can show testicular swelling or discharging scrotal sinuses.[6]

### Microscopic findings

#### Urinary tract:

**Figure 1:**
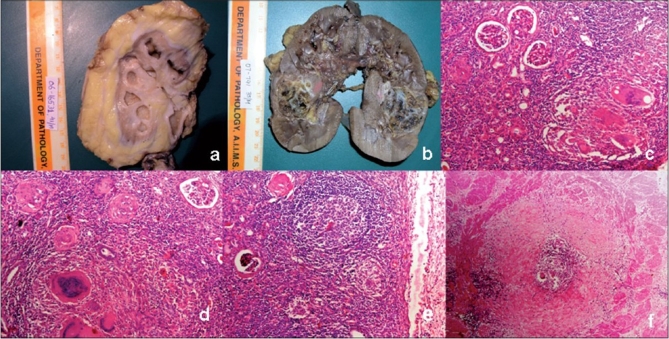
Gross photograph of kidney shows multiple necrotic areas involving the medulla and at places destroying renal calyces [Figure 1a]. Chronic TB pyelonephritis with destroyed renal calyces [Figure 1b]. Epithelioid cell granulomas with dense chronic inflammatory infiltrate in renal cortex. [Figures 1c, d, H and E, ×100]. Focal formation of lymphoid follicles [Figure 1e, H and E, ×40]. Cross-section of ureter showing ulcerated urothelium by a granulomatous process [Figure 1f, H and E, ×40]

In miliary renal TB, multiple small epitheloid granulomas with neutrophils and necrosis are seen, along with lymphocytes, mononuclear cells and plasma cell infiltration.[16] At times, the chronic inflammation may be so dense that lymphoid follicles are formed [Figure 1]. In the chronic stage, extensive fibrosis and widespread calcifications are the findings. Similar fibrotic scar can also be seen in ureteric strictures. Keratinizing squamous metaplasia may develop as a late complication in renal pelvis and may persist even after treatment of the active tuberculous lesion. This may be a potential risk factor for the development of squamous carcinoma in chronic cases.[16] Organisms may be demonstrated by standard techniques such as Ziehl-Nielsen staining.

#### Female genital tract:

**Figure 2:**
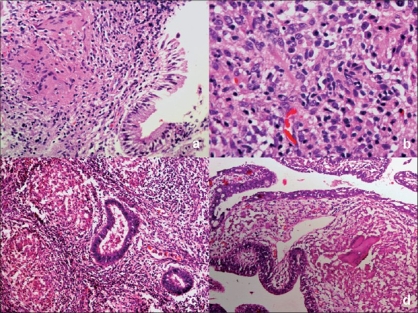
Photomicrograph demonstrates dense endometrial infiltration by plasma cells and lymphocytes [Figure 2a, H and E, ×200] with epithelioid cell granulomas [Figure 2b, H and E, ×40 and 2c, H and E, ×100] and Langhan's giant cells in endometrial tuberculosis [Figure 2d, H and E, ×100]

Similar epithelioid granulomas with Langhan's giant cells with or without necrosis are noted in functional endometrial layer with ulceration of endometrial lining at some places. Gradual destruction and loss of endometrial glands are commonly seen. Metaplasia of endometrial lining or glands is not uncommon. In the absence of granulomatous inflammation, infiltration of endometrium with plasma cells and lymphocytes is common finding[14] [Figure 2].

In immunecompromised patients, granulomas may be less well formed, organisms are readily demonstrated, and caseous necrosis is seen less frequent. Environmental mycobacteria infection, such as *M. avium-intercellulare*, may be encountered, where the lesion is more diffuse, poorly formed and the lesion consists of histiocytic cells with abundant pale cytoplasm packed with organisms (“multibacillary histiocytosis”).[9]

Similar histological features may be identified on serosal surface of fallopian tube, in ovarian stroma or parametrium. Occasionally intramyometrial granulomas are detected.

**Figure 3:**
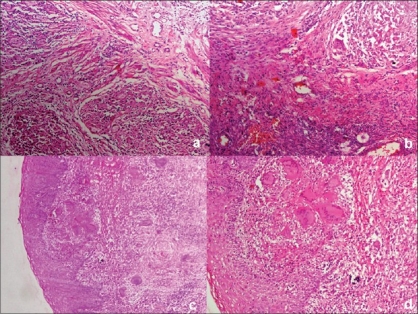
Photomicrograph shows epithelioid cell granulomas involving ovarian stroma [Figure 3a, H and E, ×40]. Granulomatous inflammation affecting myometrium [Figure 3b, H and E, ×40] and ectocervix [Figure 3c, H and E, ×40 and 3d, H and E, ×100]

However caseating granulomata alone in cervix is not diagnostic of TB [Figure 3]. And the differential diagnosis includes amoebiasis, schistosomiasis, brucellosis, tularaemia, sarcoidosis, and foreign body reaction.

Staining for acid fast bacilli is often negative. Though, isolation of the mycobacterium is the gold standard for diagnosis; culture is negative in 30% cases. Therefore, the presence of typical granulomata is sufficient for diagnosis, if other causes of granulomatous cervicitis are excluded or primary focus is identified in any other area. Histological examination of serial biopsy specimens can similarly confirm a therapeutic response.[14]

### Male genital tuberculosis

**Figure 4:**
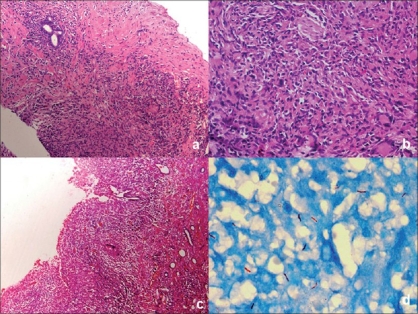
Photomicrograph of prostate trucut biopsy, showing collection of epithelioid granulomas with loss of prostatic glands and fibrosis [Figure 3a, H and E, ×40 and 4b, H and E, ×100]

The epitheloid granulomas in the prostate are usually multiple and seen in the peripheral zones [Figure 4]. Calcification is not uncommon. The scrotum shows variable degrees of fibrosis, epithelioid granulomata, inflammation, sinus tracts and focal micro-abscesses secondary to bacterial infections. The gradual development of scrotal tubercular abscess may give way and form ‘watermelon scrotum'.[6] In testes commonly epididymis is affected and shows features of granulomatous inflammation. Bilateral epididymal involvement and concomitant testicular lesion strongly suggest TB, especially in patients with evidence of TB elsewhere in the body and failure to respond to conventional antibiotic therapy.[6]

#### Urinary bladder biopsy in TB:

Usually contraindicated, unless the tubercles or the ulcers are situated away from ureteral orifice.[1]

### Diagnosis of endometrial TB

The sensitivity of endometrial biopsy or curetting in the diagnosis of TB is low (40%), as the granulomas are often focal and the functionalis layer is shed every four weeks (granulomas take two weeks to develop). So, if suspected, curettage/biopsy should be performed during the late secretory phase of the menstrual cycle. This issue has been a subject of debate and various recent studies show there is no difference of sensitivity if curetting is done in any phase of the menstrual cycle.[14] In a comparative study of sensitivity of detection of genitourinary TB, smear microscopy, histopathological examination, mycobacterial culture, nucleic acid amplification by PCR, or combination of culture and PCR were 87.5%, 82.3%, 91.6%, 96.4% and 100% respectively. While the specificity for the same were as follows, 86.36%, 84.6%, 88.88%, 100% and 100% respectively.[20,21]

However, renal TB should be suspected and treated if the tubercle bacillus is identified microscopically in a urine specimen.[22] Demonstration of acid-fast bacilli (AFB) on Ziehl-Nielsen (ZN) stain examines the patience and diligence of the pathologists. the ZN stain identifies organism at a level of 5000-10,000 bacilli/ml of sputum with a sensitivity of detection of 22-81%.[1] A thorough screening for 20 min is recommended under oil-immersion. In our routine practice, the sensitivity of detection of AFB in a classical situation is 70-75% on FNAC and 40-50% on histological sections. The detection rate falls further in liquid-based preparations. Hence definitive diagnosis requires culture of tubercle bacillus from the 3-5 am early morning voided urine samples of urine and 90% of affected patients could have a positive culture.[20] Nowadays advanced liquid culture and radiometric detection systems (BACTEC-460) or nonradiometric (Co2) detection systems (BacTAlert 3D) have increased the sensitivity and turnaround time of tubercular culture. However, in Indian studies TB culture is positive in only 30-40% of urine samples.[20] So a negative urine culture report should not rule out a possibility of TB and in these situations, polymerase chain amplification for bacterial nucleic acid provides an effective and rapid detection method for urinary TB in both pre- and post-treatment patients.[22]

### Sequel of genitourinary tuberculosis

Tuberculosis has a significant deteriorating effect on kidney function. Though often unilateral to start with, cavitary renal TB can cause renal failure in 12% patients and hypertension in 4-12% patients.[16] In one study it was reported that without surgery, the five-year survival rate of patients with renal TB was 15-42% while surgical intervention increased the 10-year survival rate to 50%.[16] If proved by selective renal artery renin estimation, nephrectomy reduces the blood pressure substantially in patients complicated with hypertension.[1] Early continuous multidrug chemotherapeutic regimens are successful in reducing mortality rate to 2.2%.[7] Of patients who die of pulmonary TB, 60% show coexistent renal TB in autopsy.[15]

Tuberculous epididymo-orchitis has a considerable effect on fertility. The sperm count and motility may be reduced due to blockage of the vas and/or secondary atrophy.[23]

Similarly, in females, 18% of the infertile females in infertility clinics suffer from TB. Amongst this, tubercular salpingitis is responsible for 72% cases and frozen pelvis in 18%.[2] Usually 25% cases of total biopsy-positive endometrial TB show synechia, leading to infertility.[2] In some studies, post-treatment conception, as low as 19% has been described, with a live birth rate of 7%. Rate of ectopic pregnancy is also considerably high after TB.[5] The development of renal amyloidosis in TB is common. This may not only cause renal dysfunction, but if not taken care of, may lead to multiple organ failures. Development of renal dysfunction in a known case of TB is therefore a strong indication for a kidney biopsy.[15]

Thus, TB leaves tons of complications on the functioning of the GU system. A proper history, strong suspicion and timely treatment can prevent the aftermath. It may be mentioned that the GU tract can also be involved by *Mycobacterium leper* (as high as 70% patients of both tuberculoid or lepromatous leprosy show renal involvement on autopsy). And nowadays as the numbers of untreatable multidrug-resistant TB are on the rise in the setting of HIV, culture and sensitivity may be applied to all patients because the modes of detection are many but none are without failure.
